# Differential Recruitment of Methyl CpG-Binding Domain Factors and DNA Methyltransferases by the Orphan Receptor Germ Cell Nuclear Factor Initiates the Repression and Silencing of *Oct4*

**DOI:** 10.1002/stem.652

**Published:** 2011-05-23

**Authors:** Peili Gu, Xueping Xu, Damien Le Menuet, Arthur C-K Chung, Austin J Cooney

**Affiliations:** a Department of Molecular and Cellular Biology, Baylor College of MedicineHouston, Texas, USA; b Department of Laboratory Medicine, Yale University of Medical SchoolNew Haven, Connecticut, USA; c INSERM, U963 Faculte de MedicineParis-Sud, 63 rue Gabriel Peri 94276, Le Kremlin Bicetre Cedex, France; d Center of Inflammatory Diseases and Molecular Therapies, The University of Hong Kong21 Sassoon Rd., Pokfulam, Hong Kong

**Keywords:** Germ cell nuclear factor, Oct4, DNA methylation, Methylated CpG binding domain, DNA methyltransferases, Embryonic stem cell

## Abstract

The pluripotency gene *Oct4* encodes a key transcription factor that maintains self-renewal of embryonic stem cell (ESC) and is downregulated upon differentiation of ESCs and silenced in somatic cells. A combination of *cis* elements, transcription factors, and epigenetic modifications, such as DNA methylation, mediates *Oct4* gene expression. Here, we show that the orphan nuclear receptor germ cell nuclear factor (GCNF) initiates *Oct4* repression and DNA methylation by the differential recruitment of methyl-CpG binding domain (MBD) and DNA methyltransferases (Dnmts) to the *Oct4* promoter. When compared with wild-type ESCs and gastrulating embryos, *Oct4* repression is lost and its proximal promoter is significantly hypomethylated in retinoic acid (RA)-differentiated *GCNF*^−/−^ ESCs and *GCNF*^−/−^ embryos. Efforts to characterize mediators of GCNF's repressive function and DNA methylation of the *Oct4* promoter identified MBD3, MBD2, and de novo Dnmts as GCNF interacting factors. Upon differentiation, endogenous GCNF binds to the *Oct4* proximal promoter and differentially recruits MBD3 and MBD2 as well as Dnmt3A. In differentiated *GCNF*^−/−^ ESCs, recruitment of MBD3 and MBD2 as well as Dnmt3A to Oct4 promoter is lost and subsequently Oct4 repression and DNA methylation failed to occur. Hypomethylation of the Oct4 promoter is also observed in RA-differentiated *MBD3*^−/−^ and *Dnmt3A*^−/−^ ESCs, but not in *MBD2*^−/−^ and *Dnmt3B*^−/−^ ESCs. Thus, recruitment of MBD3, MBD2, and Dnmt3A by GCNF links two events: gene-specific repression and DNA methylation, which occur differentially at the *Oct4* promoter. GCNF initiates the repression and epigenetic modification of *Oct4* gene during ESC differentiation. Stem Cells 2011;29:1041–1051

## INTRODUCTION

Self-renewal of Embryonic stem cell (ESC) is maintained by a core set of transcription factors: Oct4, Sox2, and Nanog, which through transcriptional regulatory loops maintain pluripotency gene expression [1, 2]. Specific mechanisms are required to disrupt this regulatory loop upon ESC differentiation to inhibit the pluripotency phenotype and allow the acquisition of a differentiated cell fate. One of the key questions in the regulation of mammalian transcription is how to regulate silencing of pluripotency genes. The *Oct4* gene is an excellent transcriptional model for understanding the regulation of pluripotency gene expression because its expression and *cis*-regulation have been well-defined in both the mouse and ESCs [2–4]. Oct4 is essential for regulation of pluripotency gene expression during early embryonic development and ESC renewal [3, [Bibr b5]–7]. Expression of the *Oct4* gene is maintained in the blastocyst and epiblast, after which it is restricted to primordial germ cells and silenced in all somatic cell lineages [8–10]. Oct4 is also expressed in ESCs and its expression is rapidly downregulated during differentiation of these cells [3, 4, 8, 11, 12].

The orphan nuclear receptor germ cell nuclear factor (GCNF) has been shown to play a central role in the repression of the *Oct4* gene upon differentiation of ESCs by binding to an evolutionarily conserved direct repeat with a zero base pair spacing (DR0) *cis*-element located in the *Oct4* promoter [8, 13–15]. GCNF is expressed during gastrulation and neurulation temporally corresponding to the in vivo repression of Oct4 [16]. Inactivation of the *GCNF* gene in mice results in embryonic lethality [8, 16, 17]. Loss of GCNF causes sustained expression of Oct4 in the early neuroectoderm and blocks the differentiation of primitive to definitive neural stem cells in vitro [18]. In ESCs, GCNF is transiently induced during early stages of retinoic acid (RA)-induced differentiation [8, 13, 19]. *GCNF*^−/−^ ESCs fail to repress Oct4 expression upon differentiation and maintain pluripotent gene expression during RA treatment [13].

Methylation of *Oct4cis*-regulatory regions and histone modifications have been reported to contribute to the silencing of the *Oct4* gene during mouse and human ESC differentiation [20–25]. DNA methylation occurs subsequent to *Oct4* repression, as loss of DNA methylation and chromatin remodeling have no effects on the initial repression of *Oct4* [20]. The regulation of *Oct4* DNA methylation is currently not well understood. The DNA methylation machinery consists of a family of DNA methyltransferases (Dnmts) (including Dnmt1, Dnmt2, Dnmt3A, Dnmt3B, and Dnmt3L), and a family of methyl-DNA binding domain (MBD) proteins [26–29]. Two MBD proteins, MBD2 and MBD3, are closely related to each other in their primary structure and belong to the MeCP1 and Mi-2/Nucleosome remodeling and deacetylase (NuRD) transcriptional repression complexes, respectively [30–33]. MBD2 binds CpG dinucleotides in a methylation-dependent manner and *MBD2* knockout (KO) mice display abnormal maternal methylation patterns [34]. In contrast, mammalian MBD3 can bind to unmethylated CpG dinucleotides [35, 36]. Genetic ablation of the *MBD3* gene leads to embryonic lethality before gastrulation, and *MBD3*^−/−^ ESCs maintain pluripotency gene expression in the absence of LIF [34, 37]. The essential function of Dnmt1 in the maintenance of DNA methylation in the mammalian cell is demonstrated by the observation that mice deficient for Dnmt1 die at midgestation with significantly reduced levels of DNA methylation [38]. The targeted disruption of *Dnmt3A* and *Dnmt3B* genes in mouse ESCs showed that both factors are essential for mouse development and methylation of the *Oct4* promoter [39–41].

To address the question of what links *Oct4* sequence-specific repression with covalent epigenetic modifications that lead to gene silencing, we investigated the molecular mechanism of *Oct4* silencing by GCNF to identify mediators of its repression function. Our results demonstrate that the interaction of GCNF with MBD2, MBD3, and Dnmt3A during differentiation initiates the repression of the *Oct4* gene and DNA methylation by means of sequential recruitment of these novel nuclear receptor corepressors.

## MATERIALS AND METHODS

### P19 and ESC Lines

*GCNF*^−/−^ and *MBD2*^−/−^ ESCs were established from mutant embryos at Embryonic 3.5-day by blastocyst outgrowth on mouse embryonic fibroblasts (MEFs). The ESCs were passaged three times off MEFs for genotyping. *MBD3*^−/−^ and *Dnmt1*^−/−^; *Dnmt3A*^−/−^, *Dnmt3B*^−/−^, and *Dnmt3A*^−/−^; as well as *Dnmt3B^−/−^* ESCs were kindly provided by Dr. Brian Hendrich [34], Dr. Rudolph Jaenisch [38], and Dr. En Li [39], respectively. Wild-type (wt) and mutant ESCs were maintained with leukemia inhibitory factor (LIF) (Chemicon, Temecula, PA, http://www.chemicon.com) on gelatinized tissue culture dishes in ESC media [13]. For the differentiation of ESCs, monolayer cultured ESCs were treated with 1 μM of all-*trans*-RA (Sigma, St. Louis, MO, http://www.sigmaaldrich.com) daily in ESC media. P19 cells were maintained in the Dulbecco's modified Eagle's media (DMEM) supplemented with 10% fetal calf serum (FCS) and 100 units of penicillin and streptomycin.

### Bisulfite Genomic Sequencing

ESC genomic DNA was extracted with Qiagen DNeasy kit (Valencia, CA, http://www.qiagen.com). Genotyping of embryos was performed as reported previously [16]. Bisulfite-modified DNA with EZ DNA methylation kit (Zymo Research, Orange, CA, http://www.zymoresearch.com) was purified and used as template for nested polymerase chain reaction (PCR). Second round PCR products were subcloned into TOPO cloning vector (Invitrogen, Carlsbad, CA, http://www.invitrogen.com) and clones were randomly picked for DNA sequencing. The sequence of the primers is listed in Supporting Information Table 1. Statistical analysis of the data used the Student's *t* test.

### Yeast Two-Hybrid Screen and Assays

DNA extracted from an amplified mouse E7 embryonic cDNA library in the yeast vector pACT2 (Clontech, Cat# 638844, Mountainview, CA, http://www.clontech.com) was cotransfected with GCNF bait plasmid pGBKT7-GCNF (ligand binding domain [LBD]) into yeast AH109 cells according to the manufacturer's protocols. First round selection was performed with 7.5 mM 3-amino-1,2,4 trizole (3-AT) and second round selection with 25 mM 3-AT. The interaction was confirmed by colony-lift and liquid β-galactosidase assays according to Clontech's protocols.

### Antibodies, GST-Pull Down, and Co-IP Assays

Anti-GCNF and anti-LRH-1 antibodies were produced by our laboratory [13, 42]. Anti-Myc, -Haemagglutin tag (HA), - Oct4, and -MBD3 antibodies were from Santa Cruz (Santa Cruz, CA, http://www.scbt.com/). Anti-Flag and -β-actin antibodies were from Sigma. Anti-MBD2 antibody for chromatin immunoprecipitation (ChIP) was from Upstate Biotechnologies (Lake Placid, NY, http://www.millipore.com) and for Westerns from Santa Cruz. Anti-Dnmt3A and -Dnmt3B monoclonal antibodies were from Imagenex (San Diego, CA, http://www.imgenex.com). Glutathione-S-transferase (GST), GST-GCNF, or GST-MBD3b proteins were expressed in *E. coli* BL21 (DE3) and purified with glutathione-agarose beads (Amersham Bioscience, Littlechalfont, England, http://www.gelifesciences.com). In vitro translated proteins were labeled with S^35^-methionine (ICN Pharmaceuticals, Costa Mesa, CA, http://www.icnpharm.com) using TNT T7 in vitro translation kit (Promega, Madison, WI, http://www.promega.com). GST-pulldown and coimmunoprecipitation (Co-IP) were performed in TBST buffer. Transfected CV-1 (simian) in Origin, and carrying the SV40 genetic material (COS-1) cell total proteins and RA-differentiated P19 cell nuclear proteins were extracted in Buffer D (25 mM Hepes pH 7.9, 150 mM KCl, 0.1 mM EDTA, 1 mM dithiothreitol (DTT), Proteinase inhibitors, and 5% glycerol). TrueBlot beads from eBioscience (San Diego, CA, http://www.ebioscience.com) were used in the co-IPs to remove the IgG, the heavy-chain of which runs very close to GCNF and MBD2.

### ChIP Assays

Undifferentiated or RA-differentiated ESCs were cross-linked with 1% formaldehyde (Sigma) and soluble chromatin was extracted and sonicated following a protocol provided by Upstate Biotechnology. Sonicated chromatin proteins were incubated with antibodies or normal IgGs and immunoprecipitated with protein A/G agarose beads (Santa Cruz). The bound DNA was eluted with sodium dodecasulphate (SDS)-proteinase K solution overnight at 65°C and extracted with phenol/CHCl_3_. PCR was performed as described [13].

### P19 Nuclear Extract Fractionation and Gel Mobility Shift Assays

P19 cell nuclear extract was prepared following the procedure described before [8] and separated through a Superose six column (HR 16/50, Pharmacia, Stockholm, Sweden, http://www.sigmaaldrich.com) by chromatography. Each 1.5 ml fraction was collected and analyzed by gel mobility shift assays [13] and western blot. The running buffer for the fast protein liquid chromatography (FPLC) was Buffer D.

### Whole-Mount In Situ hybridizations and RT-PCR

Embryos from timed matings between GCNF heterozygous mice were harvested between E8.5 and E8.75 and fixed in 4% paraformaldehyde. Whole-mount in situ hybridizations were carried out as described [13, 42]. Total RNA of ESCs was extracted with Trizole reagents (Invitrogen, Carlsbad, CA, http://www.invitrogen.com) and reverse transcribed with Advantage RT for PCR with Taq DNA polymerase (Clontech, Mountain View, CA).

## RESULTS

### Hypomethylation of Oct4 Promoter in RA-Treated *GCNF*^−/−^ ESCs

**Figure 1:**
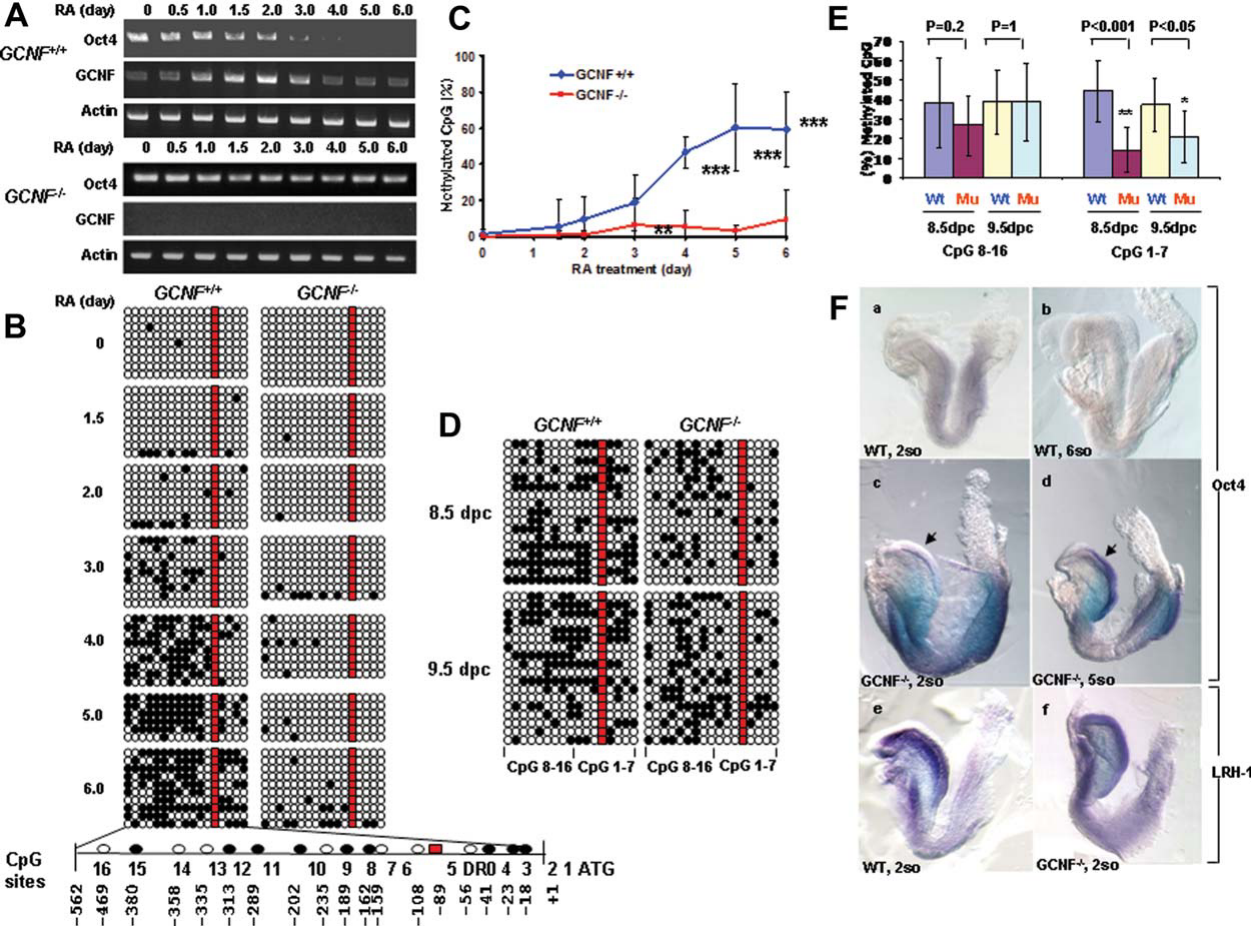
Hypomethylation of the Oct4 promoter in differentiated *germ cell nuclear factor*^−/−^ (*GCNF*^−/−^) embryonic stem cells (ESCs) and *GCNF*^−/−^ embryos at E8.5–E9.5. **(A):** Expression pattern of Oct4 and GCNF in wild-type (wt) and *GCNF*^−/−^ ESCs was detected by reverse transcriptase-polymerase chain reaction (RT-PCR). **(B):** DNA methylation profile of 16 CpG sites located in the Oct4 proximal promoter from −562 to ATG start code in wt and *GCNF*^−/−^ ESCs. The open circles represent unmethylated CpG and the black closed circles represent methylated CpG sites. The red closed square represents GCNF binding site DR0. **(C):** Comparison of percentage of methylated CpG sites in the Oct4 proximal promoter. Student's *t* test was used for the statistical analyses. **, *p* < .01; ***, *p* < .0001. **(D):** DNA methylation profile of 16 CpG sites in wt and *GCNF*^−/−^ embryos at 8.5 and 9.5 dpc. **(E):** Comparison of percentage of methylated CpG sites in the Oct4 proximal promoter by dividing the Oct4 promoter into two distal and proximal parts. **(F):** Reactivation of Oct4 gene expression in *GCNF*^−/−^ embryos detected by whole-mount in situ hybridizations. Embryos in panels a, b, and e are wt and in panels c, d, and f are *GCNF*^−/−^. Oct4 cRNA probe was used in panel a, b, c, and d; LRH-1 cRNA probe was used in panel e and f. The number of somites for each embryo is indicated in each panel. Abbreviations: DR0, direct repeat with zero base pair spacing; GCNF, germ cell nuclear factor; LRH-1, liver receptor homolog-1; mu, mutant; RA, retinoic acid; RT-PCR, reverse transcriptase-polymerase chain reaction. so, somites; wt, wild-type.

DNA methylation is required for *Oct4* gene silencing during RA-induced embryocarcinoma cells (EC) and ESC differentiation [21, 24]. Thus, we analyzed the DNA methylation profile of the *Oct4* promoter during RA-induced differentiation in *GCNF*^−/−^ ESCs when compared with wt. Repression of *Oct4* is lost in RA-differentiated *GCNF*^−/−^ ESCs (Fig. 1A) [13]. The methylation status of 16 CpG sites in the *Oct4* gene promoter was scanned at different time points of RA treatment (Fig. 1B). In undifferentiated wt ESCs and *GCNF*^−/−^ ESCs, the CpG sites were unmethylated. The onset of DNA methylation was detected when wt ESCs were treated with RA for 3 days. After 4 days, more than 50% of the CpGs were methylated, and the methylation status increased between 4 and 6 days. Interestingly, on day 3 of RA treatment, CpG methylation was not initiated in the *GCNF*^−/−^ ESCs and even at day 6 less than 10% of CpG dinucleotides were methylated (Fig. 1C). The DNA methylation analysis clearly showed that the *Oct4* promoter was hypomethylated in *GCNF*^−/−^ ESCs. When we compared the GCNF expression profile (Fig. 1A) and previous DNA binding studies [13] with CpG methylation profile (Fig. 1C), it was obvious that methylation of the Oct4 promoter occurred concomitantly with the period of elevated GCNF expression and DNA binding activity between days 1 and 3 of RA treatment in wt ESCs. Although Oct4 repression was detectable as early as day 2 of RA treatment (Fig. 1A), there was no significant (*p* < 0.1) difference in the percentage of methylated CpG dinucleotides between the wt and *GCNF*^−/−^ ESCs. After 3 days of differentiation, the percentage of methylation in *GCNF*^−/−^ ESCs was significantly lower (*p* < .01) than that observed in wt cells. At later time points of differentiation greater differences were observed (*p* < .0001) between wt and *GCNF*^−/−^ ESCs.

### Hypomethylation of the Oct4 Promoter in *GCNF*^−/−^ Embryos

Previous studies demonstrated that DNA methylation of the *Oct4* gene took place by 6.5 dpc in normal mouse embryos [24, 43]. There was loss of repression of the *Oct4* gene in many of the somatic cells in 8.5–8.75 dpc *GCNF*^−/−^ embryos [8]. Thus, the DNA methylation status of the *Oct4* promoter was analyzed in *GCNF*^−/−^ embryos. As expected, the *Oct4* promoter was heavily methylated (40% overall) in wt embryos and DNA methylation was also observed in the *GCNF*^−/−^ embryos (over all 20% at 8.5 dpc and 30% at 9.5 dpc) (Fig. 1D, 1E). However, when the *Oct4* promoter was divided into two parts: the region proximal to the GCNF DR0 element (from the first CpG to seventh CpG) and the region distal to the DR0 (from the eighth to 1sixth CpG), significant differences in DNA methylation levels were observed in the proximal region close to the DR0 between wt and *GCNF*^−/−^ embryos at both 8.5 dpc and 9.5 dpc (Fig. 1E). In contrast, there was no difference in the region distal to the DR0 between 9.5 dpc of wt and *GCNF*^−/−^ embryos. These results demonstrated that hypomethylation of the Oct4 promoter close to the GCNF binding site also occurred in *GCNF*^−/−^ embryos.

Whole-mount in situ analysis showed that the *Oct4* gene is silenced in somatic cells of wt embryos (Fig. 1F, panels a and b) and there is widespread loss of repression in somatic cells of the *GCNF*^−/−^ embryos. Careful analysis of the expression of Oct4 in *GCNF*^−/−^ embryos at multiple time points indeed showed that some of the Oct4 expression observed at the five somite stage, compared to the two somite stage, was due to re-expression of Oct4 in the neuroepithelium (Fig. 1F, panel c and d, arrowheads) as opposed to loss of repression that is observed in other regions of the embryo. Re-expression of the Oct4 gene in the neuroepithelium spatially matched the expression pattern of liver receptor homolog-1 (LRH-1), which was previously shown to regulate Oct4 expression (Fig. 1F, panel e and f) [42]. These results support the contention that loss of GCNF function leads to not only loss of repression of Oct4 but also loss of DNA methylation, allowing for a transcriptional activator, like LRH-1, to reverse the Oct4 repression and reactivate the gene.

### Identification of GCNF Interacting Factors

**Figure 2:**
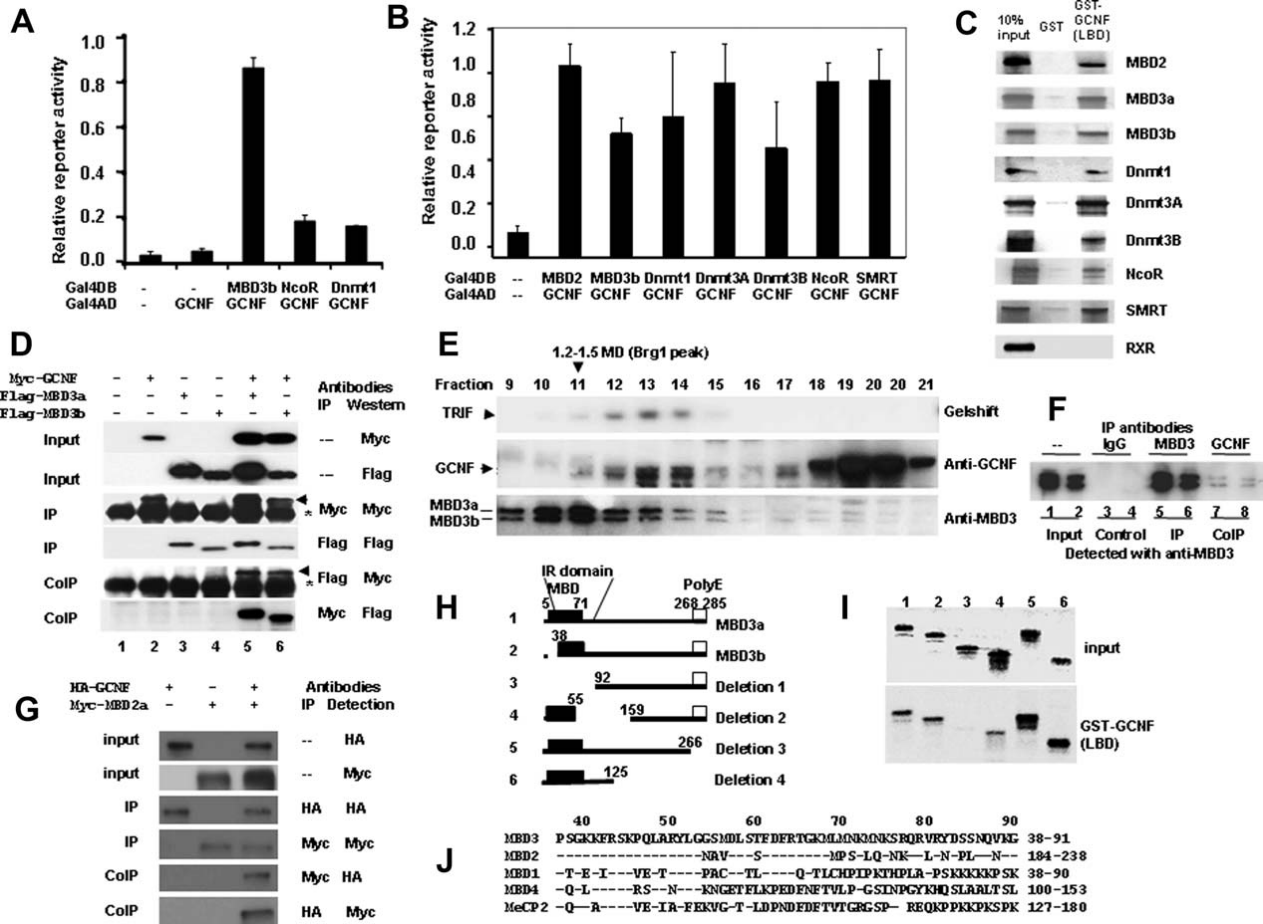
Interaction of germ cell nuclear factor (GCNF) with MBD3, MBD2, DNA methyltransferases 3A (Dnmt3A), and Dnmt3B in vitro. **(A):** Putative GCNF interaction partners identified in a Yeast two-hybrid screen. Gal4-nuclear receptor corepressor (NCoR) was used as a positive control and Gal4 DBD and Gal4 AD empty vectors were used as negative controls. **(B):** Interaction of GCNF and other DNA methylation machinery components was measured by liquid β-gal assay in yeast two-hybrid system. **(C):** Interaction of GCNF with MBDs and Dnmts was detected by GST-pulldown assay. In vitro translated NCoR was used as a positive control and RXR as a negative control. **(D):** Detection of interaction of coexpressed recombinant GCNF and MBD3 in COS-1 cells using coimmunoprecipitation (Co-IP) assays. Myc-GCNF signal is indicated with an arrow and the IgG heavy-chain is indicated with a star. **(E):** Fractions of P19 nuclear extract separated by FPLC were analyzed by gel mobility shift assays (Gelshift) and western blots with anti-GCNF and -MBD3 antibodies. **(F):** Detection of the interaction of endogenous GCNF and MBD3 in differentiated P19 cell nuclear extracts. **(G):** Interaction of GCNF with MBD2 in cotransfected COS-1 cell lysates was detected by Co-IP. **(H):** Illustration of the MBD3 deletions generated and analyzed. The number of amino acids was labeled. The black box denotes the MBD and the open box denotes for the polyglutamic acid motif (poly E). **(I):** Analysis of interaction of GCNF and different MBD3 deletions by GST-pulldown assay. **(J):** Comparison of amino acid sequence of MBDs. Abbreviations: Co-IP, coimmunoprecipitation; COS-1, CV-1 (simian) in Origin, and carrying the SV40 genetic material; DBD DNA binding domain; Dnmt, DNA methyltransferases; FPLC fast protein liquid chromatography;GCNF, Germ cell nuclear factor; GST, glutathione S transferase; IP, immunoprepitation; IR, interaction; LBD, ligand binding domain; MBD, methyl CpG binding domain; MD, MegaDaltons; NCoR, nuclear receptor corepressors; RXR, retinoid X receptor; SMRT, silencing mediator of retinioc acid and thyroid hormone receptors; TRIF, transiently retinoid induced factor.

The hypomethylation of the *Oct4* promoter in *GCNF*^−/−^ ESCs and embryos suggests that GCNF-mediated repression is directly or indirectly linked to DNA methylation of the promoter; thus, we set out to identify mediators of GCNF repression. A yeast two-hybrid screen was performed to identify GCNF interacting partners. More than 3 × 10^6^ independent mouse E7.0 embryo cDNA clones were screened with a Gal4 activation domain (AD)-GCNF LBD fusion protein as a bait. The screen identified several groups of positive colonies. One group of cDNAs shared identical sequences and encoded the short form of mouse MBD protein MBD3b. Another positive colony was identical to a region of Dnmt1 cDNA (which encodes the C-terminal of Dnmt domain). We also identified a colony encoding sequences for the corepressor NCoR, which was expected based on previous reports [8, 44, 45]. In the liquid assays, Gal4-GCNF dependent activation of β-gal reporter activity by MBD3b or Dnmt1 cotransfection was augmented to levels comparable to that observed with nuclear receptor co-repressor (NCoR) (Fig. 2A), which confirmed the interaction between GCNF, MBD3b, and Dnmt1. Considering the high conservation within the MBD family methyl-CpG binding domain (MBD) [28] and the Dnmt family in the C-terminal Dnmt catalytic domain [29], other DNA methylation components were tested for interaction with GCNF. The results demonstrated a specific interaction between MBD2, MBD3, Dnmt1, Dnmt3A, Dnmt3B, and the LBD of GCNF in vitro (Fig. 2C). In contrast, MBD1 and MBD4 yielded unreliable interaction results in these two assays. As a positive control, in vitro translated NCoR was also pulled down by GST-GCNF. As expected, in vitro translated retinoid X receptor (RXR) did not show any interaction with GST-GCNF, establishing a negative control.

### GCNF Interacts with a Subset of the MBD Family Via the MBD Domain

Association of MBD3 and MBD2 with GCNF was further corroborated in a mammalian system using Co-IP assays (Fig. 2D). When Myc-tagged GCNF and Flag-tagged MBD3a (full-length) or MBD3b were coexpressed in CV-1 (simian) in Origin, and carrying the SV40 genetic material (COS-1) cells, anti-Myc antibody coimmunoprecipitated flag-MBD3a or flag-MBD3b (lanes 5-6 in Fig. 2D, Co-IP-GCNF). Similarly, anti-Flag antibody coimmunoprecipitated Myc-GCNF (lanes 5 and 6, Co-IP-MBD3). In the untransfected (lane one) or singly transfected COS-1 cells (lanes 2–4), no Co-IP signal was observed even though the expression of the transfected protein was detected in the input and single immunoprecipitate. These results confirm that in mammalian cells GCNF can associate with MBD3. The interaction of GCNF with MBD2 was further verified by Co-IP in mammalian cells too (Fig. 2G). In cotransfected COS-1 cells, anti-HA antibodies (specific for HA-GCNF) coimmunoprecipitated Myc-MBD2, and vice versa. Thus, GCNF can interact with a subset of MBD family members, specifically MBD3 and MBD2 via the MBD domain.

However, GCNF is not expressed in COS-1 cells; rather it is transiently expressed in differentiating P19 and ESCs [8, 13]. Therefore, interaction of endogenous GCNF and MBD3 was assayed in differentiating P19 cells (Fig. 2E, 2F). First, we fractionated P19 nuclear extracts by Superose 6 gel filtration chromatography (Fig. 2E) and found that there were GCNF peaks: fraction numbers 12–14 corresponded to the transiently retinoid induced factor (TRIF) complex in gel mobility shift assays [13], and fraction numbers 18–21 were a major GCNF peak but had no DNA binding activity. MBD3a and MBD3b are both expressed in P19 cells and appeared as a major peak between fraction numbers 10 and 11. The tail of the MBD3 peak overlapped with the GCNF-TRIF complex but little MBD3 was cofractionated with the second GCNF peak. The gel filtration results suggested partial cofractionation of MBD3 with the GCNF-TRIF complex. Co-IP assays of P19 nuclear extracts also demonstrated that a small fraction of MBD3a and MBD3b were associated with GCNF and could be coimmunoprecipitated with anti-GCNF antibody (Fig. 2F, lanes 7 and 8). Under the same Co-IP conditions, no signal was detected when IgG was used. Thus, gel filtration and Co-IP results confirmed that endogenous GCNF and MBD3 interact in differentiating P19 cells.

In vitro experiments to this point confirmed interaction between GCNF's LBD and MBD3 and the catalytic domain of Dnmt family members [46]. To define the interaction domain in MBD3, N-terminal and C-terminal deletion mutants were generated (Fig. 2H) and interaction with GCNF was analyzed by GST-pulldown assays (Fig. 2I). Deletion of the C-terminus of MBD3, including the poly E domain and the coiled-coil motif (deletions 3 and 4) did not affect interaction of MBD3 with GCNF (lanes 5 and 6). However, when the MBD domain was completely (Deletion 1) or partially deleted (Deletion 2), interaction between GCNF and MBD3 was either lost (lane three) or considerably weakened (lane 4). Thus, the GCNF interaction domain in MBD3 overlaps with the MBD domain (amino acids 38–91). MBD3 is part of a discrete family of genes, which includes MeCP2 as well as MBD1, MBD2, and MBD4 [26]. Alignment of the MBD domain revealed that MBD3 shared highest amino acid sequence homology with methyl CpG binding protein 2 (MBD2) in the GCNF interaction region (especially, between amino acids 38 and 70) and relatively low homology with others in the same region (Fig. 2J) [26]. This alignment supported the Yeast two-hybrid and GST-pulldown results, which confirmed the interaction between GCNF, MBD2 and MBD3.

### GCNF-Dependent Recruitment of MBD2, MBD3, and Dnmt3A to the *Oct4* Promoter During ESC Differentiation

**Figure 3:**
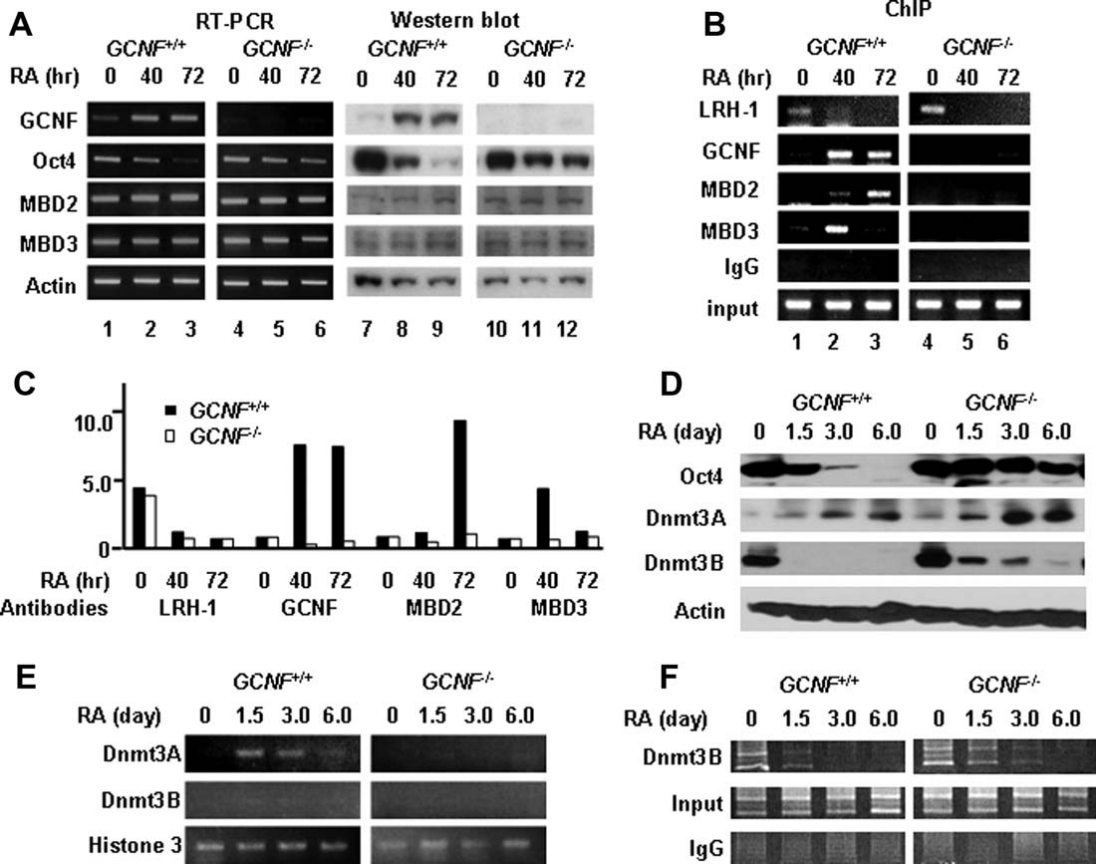
Germ cell nuclear factor (GCNF) recruits MBD2, MBD3, and DNA methyltransferases 3A (Dnmt3A) to the *Oct4* promoter in embryonic stem cells (ESCs). **(A):** Expression of GCNF, Oct4, MBD2, and MBD3 was detected in differentiated wild-type (wt) and *GCNF^−/−^* ESCs by RT-PCR and Western blot. **(B):** Binding of GCNF, MBD2, and MBD3 to *Oct4* promoter in wt and *GCNF*^−/−^ ESCs was detected by chromatin immunoprecipitation (ChIP) assay. **(C):** Quantitation of PCR signals in **(B)**. The strength of GCNF, MBD2, and MBD3 bound signals at the undifferentiated time point was set as one. The bound LRH-1 signal at 72-hour retinoic acid-differentiation was set as 1. **(D):** Expression of Oct4, Dnmt3A, and Dnmt3B in wt ESC and *GCNF*^−/−^ ESCs was detected by Western blot. **(E):** Binding of Dnmt3A to Oct4 promoter in wt ESC was detected by ChIP assay. **(F):** Binding of Dnmt3B to major satellite repeat sequence in wt and *GCNF*^−/−^ ESCs was detected by ChIP assay. Abbreviations: ChIP, chromatin immunoprecipitation; Dnmt, DNA methyltransferase; GCNF, Germ cell nuclear factor; LRH-1, liver receptor homolog-1; MBD, methyl CpG binding domain; RA, retinoic acid; RT-PCR, reverse transcriptase-polymerase chain reaction.

Direct interaction between GCNF and MBD2, MBD3, Dnmt3A, and Dnmt3B raised the question of whether GCNF recruits these factors to the Oct4 promoter. Wild-type and *GCNF*^−/−^ ESCs were cultured to compare the binding of GCNF, MBD2, and MBD3 to the *Oct4* promoter in vivo. Expression of MBD2, MBD3a, and MBD3b at the RNA and protein levels was not significantly altered upon RA treatment in either wt or *GCNF*^−/−^ ESCs (Fig. 3A). As expected, GCNF expression was upregulated (∼ 24-fold) based on quantitation of protein signals and Oct4 expression was downregulated (90%) after RA treatment; however, in *GCNF^−/−^* ESCs, the repression of Oct4 was lost and expression was maintained at high levels.

Direct binding of MBD2 and MBD3 to the *Oct4* promoter in wt and *GCNF*^−/−^ ESCs was analyzed by ChIP (Fig. 3B). To quantitatively estimate the binding signal of GCNF, MBD2, and MBD3 in wt and *GCNF*^−/−^ ESCs, ChIP signals were analyzed by densitometry and the results were plotted (Fig. 3C). To validate the ChIP samples prepared from *GCNF*^−/−^ ESCs, LRH-1 was used as a positive control to investigate binding to *Oct4* promoter [42]. Binding of LRH-1 to the *Oct4* promoter was clearly detected in *GCNF*^−/−^ ESCs, similar to wt cells. In undifferentiated ESCs no GCNF, MBD2, or MBD3 binding was detected, as expected. Upon differentiation, GCNF and MBD3 binding was enriched at the *Oct4* promoter after 40 hours of RA treatment in wt ESCs (sevenfold and fivefold increases in DNA binding were detected, respectively). At the 72-hour RA differentiation time point, GCNF protein was still detected (Fig. 3A, lane 9) and bound to the *Oct4* promoter (Fig. 3B, lane 3); meanwhile, MBD2 replaced MBD3 at the *Oct4* promoter, displaying a nine-fold increase in DNA binding. As expected, loss of GCNF binding at the *Oct4* proximal promoter resulted in the loss of recruitment of both MBD2 and MBD3 (Fig. 3B). Thus, GCNF is essential for the recruitment of MBD2 and MBD3 to the Oct4 promoter during ESC differentiation.

We investigated recruitment of the de novo Dnmts, Dnmt3A and Dnmt3B, to the *Oct4* promoter during ESC differentiation. We observed that the expression pattern of Dnmt3A and Dnmt3B was distinctly different from each other during ESC differentiation (Fig. 3D). In wt ESCs, Dnmt3A was induced upon RA treatment. In contrast, Dnmt3B was quickly repressed. In *GCNF*^−/−^ ESCs, the expression pattern of Dnmt3A was similar to wt ESCs; however, the repression of Dnmt3B was slower. Thus, Dnmt3A exhibits a similar expression pattern to GCNF, whereas Dnmt3B exhibits an expression pattern similar to Oct4. The differential expression pattern of Dnmt3A and Dnmt3B led us to question which factor plays a major role in initiating the methylation of the Oct4 promoter during ESC differentiation. We observed that Dnmt3A was preferentially recruited to the Oct4 proximal promoter at 1.5 to 3.0-day RA-treatment rather than Dnmt3B; and Dnmt3A binding was reduced at later time points (day 6) in wt ESCs. As a positive control, Dnmt3B was recruited to major satellite repeat pericentromeric chromatin in wt and *GCNF*^−/−^ ESCs and the binding signal paralleled its expression pattern (Fig. 3D, 3F), which agrees with Dnmt3B playing a major role in the methylation of CpG sites located in centromeric repeat sequences in ESCs [39, 47]. However, Dnmt3A was not recruited to major satellite repeat sequences either in wt or in *GCNF*^−/−^ ESCs during differentiation (data not shown). More importantly, the binding of Dnmt3A to the Oct4 promoter was completely absent in the *GCNF*^−/−^ ESCs (Fig. 3E). Our data suggests that GCNF preferentially recruits Dnmt3A rather than Dnmt3B to the *Oct4* promoter to initiate DNA methylation at early stages of ESC differentiation.

### DNA Methylation of the Oct4 Promoter is Defective in *MBD3*^−/−^ and *MBD2*^−/−^ ESCs

**Figure 4:**
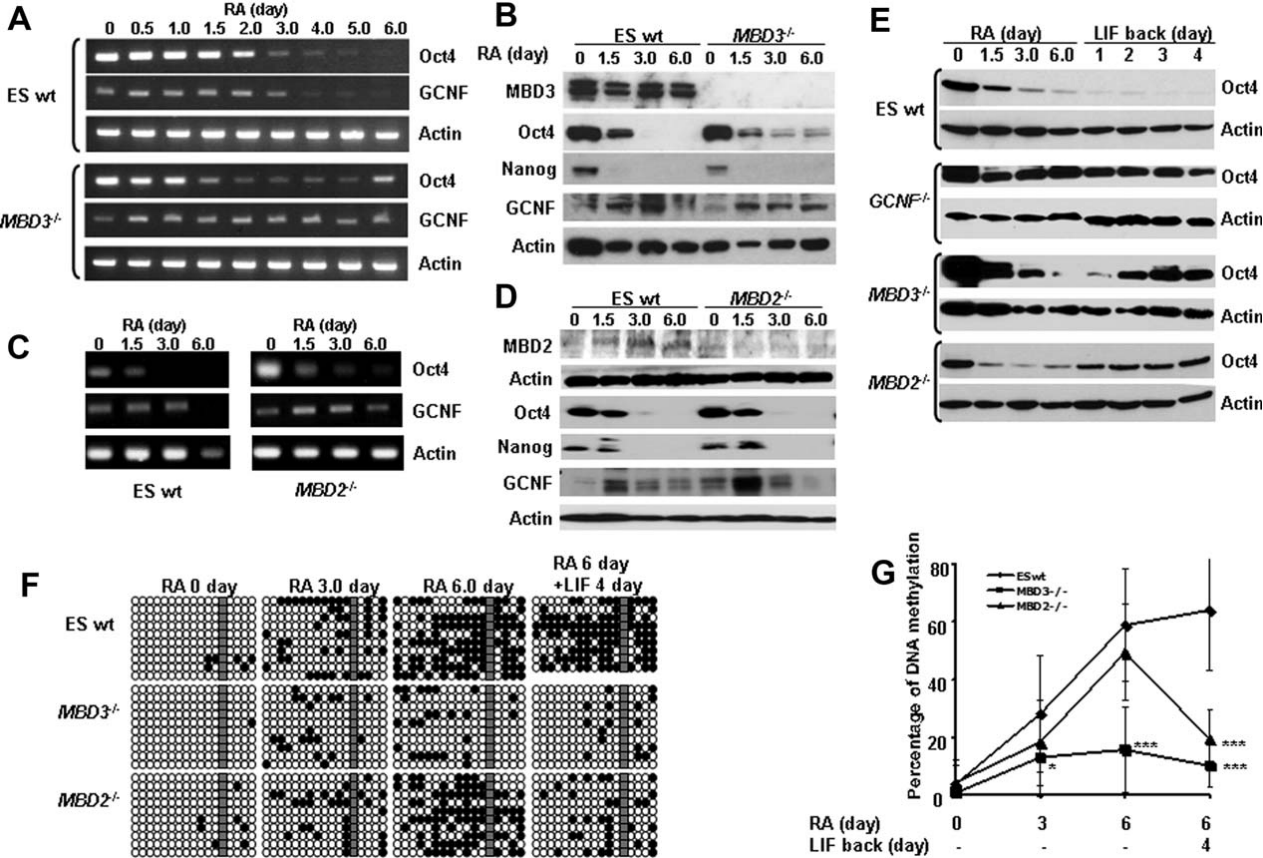
Comparison of Oct4 expression and *Oct4* promoter methylation status in *MBD3*^−/−^ and *MBD2*^−/−^ embryonic stem cells (ESCs) with wild-type (wt) ESCs and *germ cell nuclear factor*^−/−^ (*GCNF*^−/−^) ESCs. Oct4 and GCNF mRNA level **(A)** and protein level **(B)** were detected during retinoic acid (RA)-induced differentiated wt and *MBD3*^−/−^ ESCs by RT-PCR **(A)** and Western blot **(B)**. Oct4 and GCNF mRNA **(C)** and protein expression **(D)** were detected during RA-induced differentiated wt and *MBD2*^−/−^ ESCs by RT-PCR **(C)** and Western blot **(D)**. **(E):** Comparison of re-expression of Oct4 in RA-treated ESCs with LIF reactivation in wt ESCs, *GCNF*^−/−^, *MBD3*^−/−^, and *MBD2*^−/−^ ESCs. **(F):** Methylation status of *Oct4* promoter in different time point RA treatment and LIF reactivated wt ESCs, *MBD3*^−/−^, and *MBD2*^−/−^ ESCs. **(G):** Comparison of percentage of methylated CpG sites of 16 CpGs in the *Oct4* proximal promoter. Student's *t* test was used for the statistic analyses. *, *p* < .05; **, *p* < .01; ***, *p* < .0001. Abbreviations: ESC, embryonic stem cell; GCNF, germ cell nuclear factor; LIF, leukemia inhibitory factor; MBD, methyl CpG binding domain; RA, retinoic acid; wt, wild-type; RT-PCR, reverse transcriptase-polymerase chain reaction.

To further investigate the role of the interaction of GCNF with MBD2 and MBD3 in ESCs, the expression of GCNF and Oct4 was compared in wt ESCs to *MBD3*^−/−^ (Fig. 4A, 4B) and *MBD2*^−/−^ ESCs (Fig. 4C, 4D). Oct4 expression was maintained at a low level in the *MBD3*^−/−^ ESCs during RA-treatment, which implied that it was not properly silenced. This pattern was different from that of the *GCNF*^−/−^ ESCs, where Oct4 repression is lost and expression is maintained at comparable levels to undifferentiated cells (Figs. 1A, 3D). However, another pluripotency factor, Nanog, exhibited the same repression pattern in *MBD3*^−/−^ ESCs as in wt ESCs (Fig. 4B). Surprisingly, GCNF was induced in *MBD3*^−/−^ ESCs and maintained at later stages of RA treatment (days 3–6). These observations support our hypothesis that loss of MBD3 results in loss of *Oct4* silencing during RA-induced ESC differentiation. In *MBD2*^−/−^ ESCs, we observed that Oct4, Nanog, and GCNF showed the same expression profile at the mRNA and protein levels as in wt cells (Fig. 4C, 4D). Thus, we were unable to determine the role of MBD2 recruitment to the *Oct4* promoter. To further understand the different functions of MBD2 and MBD3 in the regulation of Oct4 expression in ESCs, we designed an RA-differentiation and LIF rescue experiment to study silencing and methylation status of the *Oct4* promoter in *MBD3*^−/−^ and *MBD2*^−/−^ ESCs. As expected, Oct4 expression in wt ESCs is sufficiently silenced because there was no reactivation of Oct4, and methylation of the Oct4 promoter was maintained (60%, Fig. 4F, 4G) with re-addition of LIF after 6 days RA-treatment in wt ESC. Surprisingly, although Oct4 repression was observed in *MBD3*^−/−^ and *MBD2*^−/−^ ESCs, readministration of LIF restored Oct4 expression in both mutant ESC lines (Fig. 4E). Loss of MBD3 causes deficiency of DNA methylation of the Oct4 promoter in RA-differentiated ESCs (18% in *MBD3*^−/−^ vs. 60% in wt at 6-day RA treatment), despite the fact that GCNF exhibited higher expression in *MBD3*^−/−^ ESCs (Fig. 4B) at this time point. The hypomethylation defect observed in *MBD3*^−/−^ ESCs was reiterated when LIF was added back after RA treatment. Deletion of MBD2 did not affect the DNA methylation status of the Oct4 promoter during RA-induced differentiation. Surprisingly, methylation of the *Oct4* promoter was dramatically reduced from 55% to 20% in *MBD2*^−/−^ ESCs with readdition of LIF, indicating that *Oct4* silencing was affected. The DNA methylation profiles of the Oct4 promoter in *MBD3*^−/−^ and *MBD2*^−/−^ ESCs (Fig. 4F, 4G) reinforced the separate functions of MBD3 and MBD2 in the repression and silencing of Oct4. These data suggest that MBD2 plays a role in maintenance of *Oct4* silencing, while MBD3 is essential for initiating DNA methylation and Oct4 silencing. Importantly, both factors function in a GCNF-dependent manner.

### Defective DNA Methylation of the *Oct4* Promoter in Different Dnmt Mutant ESCs

**Figure 5:**
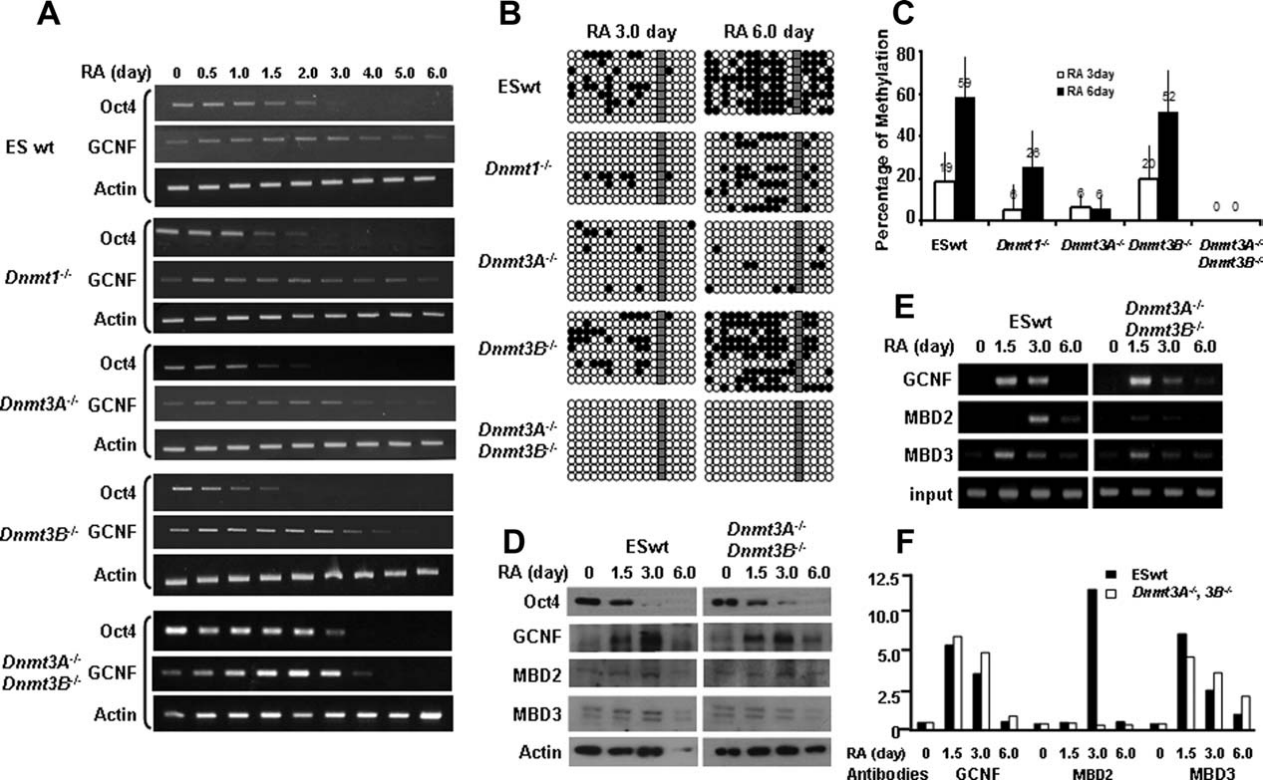
Oct4 expression and *Oct4* promoter methylation status in different DNA methyltransferases (Dnmt) mutant embryonic stem cells (ESCs). **(A):** Oct4 and germ cell nuclear factor (GCNF) mRNA level were detected during retinoic acid (RA)-induced differentiated wild-type (wt) ESCs, *Dnmt1*^−/−^, *Dnmt3A*^−/−^, *Dnmt3B*^−/−^, and *Dnmt3A^−/−^* and *Dnmt3B^−/−^* double mutant ESCs by RT-PCR. **(B):** Methylation status of *Oct4* promoter in wt ESC and Dnmt mutant ESCs treated with RA for 3 and 6 days. **(C):** Quantitation of methylated CpG sites of 16 CpGs in the *Oct4* proximal promoter. Student's *t* test was used for the statistic analyses. **, *p* < .01; ***, *p* < .0001. **(D):** Expression of Oct4, GCNF, MBD2, and MBD3 in wt ESC and *Dnmt3A*^−/−^ and *Dnmt3B*^−/−^ double mutant ESCs. **(E):** Recruitment of MBD2 and MBD3 by GCNF in *Dnmt3A*^−/−^ and *Dnmt3B*^−/−^ double mutant ESCs was analyzed by chromatin immunoprecipitation assay. **(F):** Quantitation of binding of GCNF, MBD2, and MBD3 to Oct4 promoter in *Dnmt3A*^−/−^ and *Dnmt3B*^−/−^ ESCs. The strength of GCNF, MBD2, and MBD3 bound signals at the undifferentiated time point was set as 1. Abbreviations: Dnmt, DNA methyltransferase; ESC, embryonic stem cell; GCNF, germ cell nuclear factor; MBD, methyl CpG binding domain; RA, retinoic acid; wt, wild-type; RT-PCR, reverse transcriptase-polymerase chain reaction.

In light of the direct interaction between GCNF and Dnmt3A in ESCs (Fig. 3E), we further compared the expression of Oct4 and the DNA methylation status of its promoter in different Dnmt mutant ESC lines. GCNF induction and Oct4 repression were not altered in all Dnmt mutant cell lines, including *Dnmt1*^−/−^, *Dnmt3A*^−/−^, *Dnmt3B*^−/−^, as well as *Dnmt3A*^−/−^ and *Dnmt3B*^−/−^ (Fig. 5A). Their DNA methylation status are depicted in Figure 5B and quantitated in Figure 5C. *Dnmt1* disruption leads to a reduction of methylated CpG percentage to approximately half that of wt ESCs at 6 days of treatment, and is consistent with the role of Dnmt1 in mediating DNA methylation during DNA replication. Interestingly, disruption of *Dnmt3A* alone resulted in almost complete loss of DNA methylation of Oct4 promoter, whereas deletion of *Dnmt3B* did not affect DNA methylation of Oct4 promoter (19% vs. 20% at day 3 and 52% vs. 59% in wt ESCs at day 6). As previously reported, double KO of *Dnmt3A* and *Dnmt3B* caused a complete loss of DNA methylation of Oct4 promoter [40]. The DNA methylation profiles support our previous finding that GCNF preferentially interacts with Dnmt3A rather than Dnmt3B in differentiated ESCs.

### Recruitment of MBD3 Is Independent of De Novo DNA Methylation

To address whether the differential recruitment of MBD3 and MBD2 is dependent on DNA methylation, we analyzed Oct4 repression and DNA methylation in de novo DNA methylation deficient ESCs. Although DNA methylation is lost in *Dnmt3A*^−/−^ and *Dnmt3B*^−/−^ ESCs (Fig. 5B), the initial repression of Oct4 exhibits the same temporal pattern as wt ESCs (Fig. 5A). Western blot analysis confirmed the induction of GCNF and maintenance of Oct4 repression. Furthermore, MBD3 and MBD2 expression is maintained in the *Dnmt3A*^−/−^ and *Dnmt3B*^−/−^ ESCs (Fig. 5C). The recruitment of MBD2 and MBD3 to the *Oct4* promoter was detected in Dnmt3A and Dnmt3B double KO ESCs (Fig. 5E) and quantitated in Figure 5F. Binding of GCNF, MBD2, and MBD3 to the *Oct4* promoter was confirmed between 0 and 3 days of RA-treatment; interestingly, neither MBD2 nor MBD3 is bound to the Oct4 promoter at day 6. GCNF binding to the *Oct4* promoter was observed in the *Dnmt3A*^−/−^ and *Dnmt3B*^−/−^ ESCs in a pattern similar to wt cells. Recruitment of MBD2 to the *Oct4* promoter was lost in the *Dnmt3A*^−/−^ and *Dnmt3B*^−/−^ ESCs; however, the GCNF-dependent binding of MBD3 was maintained in *Dnmt3A*^−/−^ and *Dnmt3B*^−/−^ ESCs. These results demonstrate that the recruitment of MBD3 is independent of de novo DNA methylation, but requires GCNF. In contrast, MBD2 recruitment is dependent on both CpG methylation and the binding of GCNF. Finally, it is clear that disruption of Dnmt3A alone causes hypomethylation of *Oct4* promoter; however, this defect has no bearing on the GCNF-dependent recruitment of MBD3 to the *Oct4* promoter.

## DISCUSSION

This study demonstrates that repression of *Oct4* is mediated by recruitment of novel NCoRs CpG binding proteins MBD3 and MBD2 and the de novo Dnmt, Dnmt3A, to the *Oct4* promoter via direct interactions with the orphan nuclear receptor GCNF during RA-induced differentiation of mouse ESCs. The time course of differential recruitment of MBD2 and MBD3 to the *Oct4* promoter correlates with the DNA methylation status of the promoter (Fig. 1). Although MBD2 and MBD3 are structurally closely related to each other, KO mouse models for each gene clearly establish that they are not functionally redundant. MBD2 was shown to bind methylated DNA and interact with MBD3 in a NuRD repression complex [30, 48] or with Sin3A in a Sin3A repression complex [49]. The *MBD3* KO and *MBD3*^−/−^ ESCs demonstrate an important role for the MBD3-NuRD complex during the early embryonic development and ESC differentiation, respectively [34, 37]. The dependence of MBD2 and independence of MBD3 recruitment to the *Oct4* promoter on DNA methylation underscores the mechanistic differences between these factors and their complexes. The differential dependence of MBD3 and MBD2 on DNA methylation may provide a rationale for the significant differences observed in the phenotypes of the two KO models. One could speculate that MBD3 being important for the repression of genes such as *Oct4* would display dramatic defects upon inactivation due to loss of repression of target genes. In contrast, inactivation of MBD2 would have less acute effects, because it appears to be involved in maintaining gene silencing rather than repression. Our findings clearly define a role for MBD3 at the molecular level in the repression of *Oct4* expression during ESC differentiation. The time-delay between *Oct4* repression and DNA methylation also indicates that *Oct4* repression occurs at the unmethylated stage, in which GCNF binding and interaction with MBD3 take place. Oligomerization of GCNF in differentiated P19 and ESCs likely accelerates the recruitment of MBD3 and spreading of the repression complex throughout the *Oct4* promoter [50]. Oligomerization of GCNF may also facilitate the simultaneous recruitment of de novo Dnmt, Dnmt3A, to CpG sites to initiate methylation of the promoter. Additionally, promoter occupation by GCNF and MBD3 may protect from transactivator binding (e.g., LRH-1) and facilitate subsequent histone deacetylation, histone methylation, DNA methylation, and recruitment of MBD2, leading ultimately to gene silencing [20]. Thus, direct recruitment of MBD2 and MBD3 represents a novel repression model for nuclear receptors and may link epigenetic modification to gene-specific repression by nuclear receptors. Our results are also consistent with the recent observations that showed MBD2 and MBD3 belong to distinct NuRD repression complexes [51].

It has been reported that GCNF interacts with Dnmt3A and Dnmt3B via the proline tryptophan tryptophan proline (PWWP) domain and the catalytic C-terminus in vitro [46]. We also found that GCNF can directly interact with the C-terminus of Dnmt family members, Dnmt1, Dnmt3A, and Dnmt3B in vitro; however, in vivo GCNF preferentially recruits Dnmt3A to *Oct4* promoter (Fig. 3). It is consistent with a previous study using the Dnmt KO ESCs, which showed that although both Dnmt3A and Dnmt3B play a role in the repression of Oct4, Dnmt3A plays the dominant role [40]. Dnmt3B protein is rapidly downregulated with RA-treatment, thus there is little overlap between the periods of expression of Dnmt3B and GCNF. Dnmt3A induction parallels GCNF expression and overlap with each other from 1.5 to 4 days of RA-treatment, which provides a temporal window of overlap that allows for interaction (Fig. 5).

**Figure 6:**
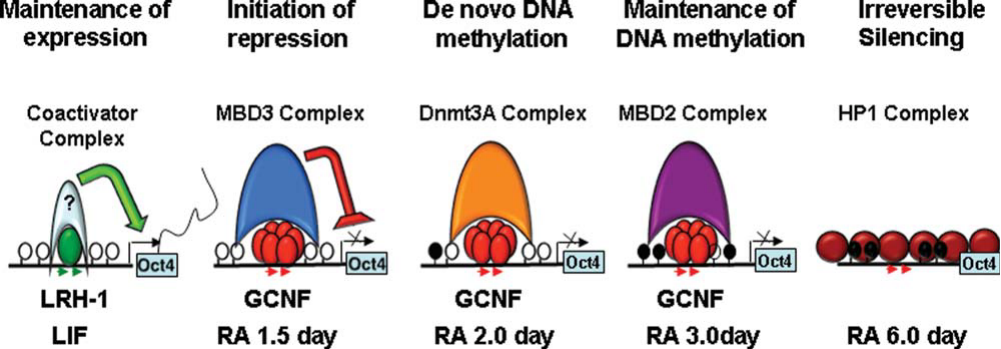
Model of *Oct4* gene repression and silencing initiated by germ cell nuclear factor (GCNF)-dependent recruitment of MBD2, MBD3, and DNA methyltransferases 3A (Dnmt3A). The *Oct4* promoter with a DR0 and unmethylated CpG sites is activated by LRH-1 under the control of LIF. At the beginning of retinoic acid (RA) induction (1.5 days), induced expression of GCNF hexamer replaces LRH-1 binding at the DR0 site. GCNF recruits MBD3 complex to unmethylated CpG sites and Oct4 repression is initiated. Once de novo DNA methylation is triggered through direct recruitment of Dnmt3A to the *Oct4* promoter by GCNF-MBD3 or MBD2/3 complexes recruited to CpG sites and silencing of Oct4 gene occurs (days 1.5–3.0). At late stages of RA-induced differentiation (days 3–6), the expression of GCNF is downregulated and the MBD2 and MBD3 complexes are no longer bound to the *Oct4* promoter but DNA methylation is maintained and *Oct4* gene is completely silenced (day 6). HP1 complex maybe replaced by GCNF/DNA methylation complex to bind to methylated *Oct4* promoter region. Abbreviations: Dnmt, DNA methyltransferase; DR0, direct repeat with zero base pair spacing; GCNF, germ cell nuclear factor; HP1, heterchromatin protein 1; LIF, leukemia inhibitory factor; LRH-1, liver receptor homolog-1; MBD3, methyl CpG binding domain 3; RA, retinoic acid.

Repression and silencing of *Oct4* is a multistep process requiring various epigenetic covalent modifications (DNA and histones) [20, 22-25, 52, 53]. Each modification requires specific factors and complexes be brought to the promoter to facilitate efficient repression. Loss of any one of these factors or failure to recruit a repressor complex will lead to loss of proper silencing; however, repression of *Oct4* can still be observed [20, 52]. Case in point are Dnmt 3A and Dnmt3B, which are required for the de novo methylation of the Oct4 gene and its silencing (Fig. 6) [20, 52]; however, Oct4 expression is still repressed when the double KO ESCs are treated with RA. The same phenomenon occurs in *G9*α^−/−^ [20], *MBD3*^−/−^ (Fig. 4), and *Dnmt3A*^−/−^ ESCs (Fig. 5). Repression of *Oct4* still occurs in these mutant ESCs most likely because GCNF is still expressed and expression of activators, like LRH-1, are repressed [42]. The binding of GCNF to the *Oct4* promoter is sufficient to displace activators, like LRH-1, and trigger a transition from activation and to repression. Thus, inactivation of individual corepressors such as MBD2, MBD3, Dnmt3A, and G9a that mediate repression and silencing of *Oct4* does not lead to loss of repression, rather they result in hypomethylation of the *Oct4* promoter and loss of silencing of *Oct4* expression (Figs. 4, 5). Only when the initiator and coordinator of *Oct4* repression, GCNF, is inactivated, both *Oct4* repression and DNA methylation are lost and Oct4 expression is maintained at higher levels than in the mutant corepressor ESC lines (Figs. 3–5). Recently, it was found that G9a can recruit Dnmt3a and Dnmt3b to the Oct4 promoter through direct interaction between the G9a ankaryn (ANK) domain and the Dnmt MTD domain [54]. We found abnormal Dnmt3 expression in *G9a*^−/−^ ESCs. It will be interesting to further understand the relationship between GCNF-dependent repression and histone modification by G9a and DNA methylation by Dnmt3a/Dnmt3b in different genetically deficient ESCs.

## SUMMARY

Our results have established GCNF as an important initiator of *Oct4* gene repression and silencing, and we propose the following model as the important key steps in *Oct4* repression in ESCs (Fig. 6). In undifferentiated and early differentiating ESCs transactivators, such as LRH-1 bind to the DR0 site in the *Oct4* promoter and sites in the proximal enhancer to maintain *Oct4* gene expression [42]. Upon induction with RA, GCNF hexamer displaces LRH-1 from the DR0, causing passive loss of activation. Concomitant with binding to the DR0, GCNF recruits MBD3 and likely the Mi-2/NuRD complex, which binds to unmethylated CpGs to initiate active repression of Oct4. Subsequently, de novo DNA methylation occurs with the recruitment of Dnmt3A. Later events, recruitment of Dnmt3A and MBD2 by GCNF to bind to methylated CpG dinucleotides, lead to an increase in DNA methylation. Corecruitment of MBD3, MBD2, and Dnmt3A to the Oct4 promoter can be detected in ESCs between 36 and 72 hours. The apparent corecruitment could reflect asynchrony in the ESC cultures or may be because of direct interactions between MBD2 and MBD3 [30], which cannot be ruled out. Alternatively, simple intermediate complexes containing both MBD2 and MBD3 maybe present simultaneously on the *Oct4* promoter as depicted in Figure 6. In addition, the differentiating ESCs undergo proliferation thus Dnmt1 is required to maintain the fully methylated status of CpG dinucleotides after DNA replication. Subsequently, GCNF expression itself is repressed but *Oct4* DNA methylation is maintained, reflective of true gene silencing as MBD2 complexes and/or other repression complexes, such as the HP1 complex are docked on the *Oct4* promoter [55]. This model is the first to define the initiation steps of *Oct4* gene repression and DNA methylation mediated by GCNF and proposes differential functions for MBD3 before de novo DNA methylation and MBD2 after DNA methylation.

## References

[1] Boyer LA, Lee TI, Cole MF (2005). Core transcriptional regulatory circuitry in human embryonic stem cells. Cell.

[2] Boiani M, Scholer HR (2005). Regulatory networks in embryo-derived pluripotent stem cells. Nat Rev Mol Cell Biol.

[3] Pan GJ, Chang ZY, Scholer HR (2002). Stem cell pluripotency and transcription factor Oct4. Cell Res.

[4] Chambers I (2004). The molecular basis of pluripotency in mouse embryonic stem cells. Cloning Stem Cells.

[b5] Pesce M, Scholer HR (2000). Oct-4: Control of totipotency and germline determination. Mol Reprod Dev.

[6] Scholer HR, Hatzopoulos AK, Balling R (1989). A family of octamer-specific proteins present during mouse embryogenesis: Evidence for germline-specific expression of an Oct factor. EMBO J.

[7] Okumura-Nakanishi S, Saito M, Niwa H (2005). Oct-3/4 and Sox2 regulate Oct3/4 gene in ES cells. J Biol Chem.

[8] Fuhrmann G, Chung AC, Jackson KJ (2001). Mouse germline restriction of Oct4 expression by germ cell nuclear factor. Dev Cell.

[9] Nichols J, Zevnik B, Anastassiadis K (1998). Formation of pluripotent stem cells in the mammalian embryo depends on the POU transcription factor Oct4. Cell.

[10] Kehler J, Tolkunova E, Koschorz B (2004). Oct4 is required for primordial germ cell survival. EMBO Rep.

[11] Niwa H, Miyazaki J, Smith AG (2000). Quantitative expression of Oct-3/4 defines differentiation, dedifferentiation or self-renewal of ES cells. Nat Genet.

[12] Schoorlemmer J, van Puijenbroek A, van Den Eijnden M (1994). Characterization of a negative retinoic acid response element in the murine Oct4 promoter. Mol Cell Biol.

[13] Gu P, LeMenuet D, Chung AC (2005). Orphan nuclear receptor GCNF is required for the repression of pluripotency genes during retinoic acid-induced embryonic stem cell differentiation. Mol Cell Biol.

[14] Cooney AJ, Lee CT, Lin SC (2001). Physiological function of the orphans GCNF and COUP-TF. Trends Endocrinol Metab.

[15] Yan ZH, Medvedev A, Hirose T (1997). Characterization of the response element and DNA binding properties of the nuclear orphan receptor germ cell nuclear factor/retinoid receptor-related testis-associated receptor. J Biol Chem.

[16] Chung AC, Katz D, Pereira FA (2001). Loss of orphan receptor germ cell nuclear factor function results in ectopic development of the tail bud and a novel posterior truncation. Mol Cell Biol.

[17] Lan ZJ, Chung AC, Xu X (2002). The embryonic function of germ cell nuclear factor is dependent on the DNA binding domain. J Biol Chem.

[18] Akamatsu W, DeVeale B, Okano H (2009). Suppression of Oct4 by germ cell nuclear factor restricts pluripotency and promotes neural stem cell development in the early neural lineage. J Neurosci.

[19] Lei W, Hirose T, Zhang LX (1997). Cloning of the human orphan receptor germ cell nuclear factor/retinoid receptor-related testis-associated receptor and its differential regulation during embryonal carcinoma cell differentiation. J Mol Endocrinol.

[20] Feldman N, Gerson A, Fang J (2006). G9a-mediated irreversible epigenetic inactivation of Oct-3/4 during early embryogenesis. Nat Cell Biol.

[21] Ben-Shushan E, Pikarsky E, Klar A (1993). Extinction of Oct-3/4 gene expression in embryonal carcinoma x fibroblast somatic cell hybrids is accompanied by changes in the methylation status, chromatin structure, and transcriptional activity of the Oct-3/4 upstream region. Mol Cell Biol.

[22] Tsuji-Takayama K, Inoue T, Ijiri Y (2004). Demethylating agent, 5-azacytidine, reverses differentiation of embryonic stem cells. Biochem Biophys Res Commun.

[23] Simonsson S, Gurdon J (2004). DNA demethylation is necessary for the epigenetic reprogramming of somatic cell nuclei. Nat Cell Biol.

[24] Gidekel S, Bergman Y (2002). A unique developmental pattern of Oct-3/4 DNA methylation is controlled by a *cis*-demodification element. J Biol Chem.

[25] Deb-Rinker P, Ly D, Jezierski A (2005). Sequential DNA methylation of the Nanog and Oct-4 upstream regions in human NT2 cells during neuronal differentiation. J Biol Chem.

[26] Hendrich B, Bird A (1998). Identification and characterization of a family of mammalian methyl-CpG binding proteins. Mol Cell Biol.

[27] Bird A (1999). DNA methylation de novo. Science.

[28] Hendrich B, Bird A (2000). Mammalian methyltransferases and methyl-CpG-binding domains: Proteins involved in DNA methylation. Curr Top Microbiol Immunol.

[29] Chen T, Li E (2004). Structure and function of eukaryotic DNA methyltransferases. Curr Top Dev Biol.

[30] Zhang Y, Ng HH, Erdjument-Bromage H (1999). Analysis of the NuRD subunits reveals a histone deacetylase core complex and a connection with DNA methylation. Genes Dev.

[31] Wade PA, Gegonne A, Jones PL (1999). Mi-2 complex couples DNA methylation to chromatin remodelling and histone deacetylation. Nat Genet.

[32] Simmen MW, Leitgeb S, Charlton J (1999). Nonmethylated transposable elements and methylated genes in a chordate genome. Science.

[33] Elliott K, Sakamuro D, Basu A (1999). Bin1 functionally interacts with Myc and inhibits cell proliferation via multiple mechanisms. Oncogene.

[34] Hendrich B, Guy J, Ramsahoye B (2001). Closely related proteins MBD2 and MBD3 play distinctive but interacting roles in mouse development. Genes Dev.

[35] Jorgensen HF, Bird A (2002). MeCP2 and other methyl-CpG binding proteins. Ment Retard Dev Disabil Res Rev.

[36] Saito M, Ishikawa F (2002). The mCpG-binding domain of human MBD3 does not bind to mCpG but interacts with NuRD/Mi2 components HDAC1 and MTA2. J Biol Chem.

[37] Kaji K, Caballero IM, MacLeod R (2006). The NuRD component Mbd3 is required for pluripotency of embryonic stem cells. Nat Cell Biol.

[38] Li E, Bestor TH, Jaenisch R (1992). Targeted mutation of the DNA methyltransferase gene results in embryonic lethality. Cell.

[39] Okano M, Bell DW, Haber DA (1999). DNA methyltransferases Dnmt3a and Dnmt3b are essential for de novo methylation and mammalian development. Cell.

[40] Li JY, Pu MT, Hirasawa R (2007). Synergistic function of DNA methyltransferases Dnmt3a and Dnmt3b in the methylation of Oct4 and Nanog. Mol Cell Biol.

[41] Chen T, Ueda Y, Dodge JE (2003). Establishment and maintenance of genomic methylation patterns in mouse embryonic stem cells by Dnmt3a and Dnmt3b. Mol Cell Biol.

[42] Gu P, Goodwin B, Chung AC (2005). Orphan nuclear receptor LRH-1 is required to maintain Oct4 expression at the epiblast stage of embryonic development. Mol Cell Biol.

[43] Yamazaki Y, Fujita TC, Low EW (2006). Gradual DNA demethylation of the Oct4 promoter in cloned mouse embryos. Mol Reprod Dev.

[44] Yan Z, Kim YS, Jetten AM (2002). RAP80, a novel nuclear protein that interacts with the retinoid-related testis-associated receptor. J Biol Chem.

[45] Yan Z, Jetten AM (2000). Characterization of the repressor function of the nuclear orphan receptor retinoid receptor-related testis-associated receptor/germ cell nuclear factor. J Biol Chem.

[46] Sato N, Kondo M, Arai K (2006). The orphan nuclear receptor GCNF recruits DNA methyltransferase for Oct-3/4 silencing. Biochem Biophys Res Commun.

[47] Lehnertz B, Ueda Y, Derijck AA (2003). Suv39h-mediated histone H3 lysine 9 methylation directs DNA methylation to major satellite repeats at pericentric heterochromatin. Curr Biol.

[48] Tatematsu KI, Yamazaki T, Ishikawa F (2000). MBD2-MBD3 complex binds to hemi-methylated DNA and forms a complex containing DNMT1 at the replication foci in late S phase. Genes Cells.

[49] Boeke J, Ammerpohl O, Kegel S (2000). The minimal repression domain of MBD2b overlaps with the methyl-CpG-binding domain and binds directly to Sin3A. J Biol Chem.

[50] Gu P, Morgan DH, Sattar M (2005). Evolutionary trace-based peptides identify a novel asymmetric interaction that mediates oligomerization in nuclear receptors. J Biol Chem.

[51] Le Guezennec X, Vermeulen M, Brinkman AB (2006). MBD2/NuRD and MBD3/NuRD, two distinct complexes with different biochemical and functional properties. Mol Cell Biol.

[52] Jackson M, Krassowska A, Gilbert N (2004). Severe global DNA hypomethylation blocks differentiation and induces histone hyperacetylation in embryonic stem cells. Mol Cell Biol.

[53] Xi S, Geiman TM, Briones V (2009). Lsh participates in DNA methylation and silencing of stem cell genes. Stem Cells.

[54] Epsztejn-Litman S, Feldman N, Abu-Remaileh M (2008). De novo DNA methylation promoted by G9a prevents reprogramming of embryonically silenced genes. Nat Struct Mol Biol.

[55] Loh YH, Zhang W, Chen X (2007). Jmjd1a and Jmjd2c histone H3 Lys 9 demethylases regulate self-renewal in embryonic stem cells. Genes Dev.

