# Supplementary material for: Differential Recruitment of Methyl CpG-Binding Domain Factors and DNA Methyltransferases by the Orphan Receptor Germ Cell Nuclear Factor Initiates the Repression and Silencing of Oct4
Source: Stem Cells. 2011 May 23;29(7):1041–51. doi: 10.1002/stem.652 (PMC3468724; doi:10.1002/stem.652)
Supplement: Supplementary file 1 [file stem0029-1041-SD1.doc]

Table 1: Sequences of RT- PCR and ChIP primers

| Primers for RT-PCR | |
| --- | --- |
| Actin | F:GGCCCAGAGCAAGAGAGGTATCC  R:ACGCACGATTTCCCTCTCTCAGC |
| GCNF | F:CTGAACAACGAACCTGTCTC  R:ACATGACACAGTTCTTGTCAC |
| Oct4 | F:GGCGTTCTCTTTGGAAAGGTGTTC  R:CTCGAACCACATCCTTCTCT |
| MBD2 | F:GGCTACAAGGACTTAGCGCAT  R:CTTGCGTACTTGTTGGACTCGC |
| MBD3 | F:AAGCCTGACCTGAACACCG  R:CAGGGCACCTGTGCTACACTC |
| Primers for Nested PCR of Bisulfite treated Oct4 proximal promoter | |
| First round PCR primers | F: AAATTTTGGAGGATTGGAGGTGTAATGG  R: CTAAACCCAATCCAACCTAAAATCCACA |
| Second round PCR primers | F: ATATTGGGTTTATTTATATTTAGGATTTTA  R: AAATCTAAAACCAAATATCCAACCATAA |
| ChIP PCR primers for Satellite repeat sequences | |
| Major satellites repeat sequence | (MajF1) 5-GACGACTTGAAAAATGACGAAATC-3 and  (MajR1) 5-CATATTCCAGGTCCTTCAGTGTGC-3;  size (308, 542, 776bp) |
| Minor satellites repeat sequence | (MinF1) 5-CATGGAAAATGATAAAAACC-3 and  (MinR1) 5-CATCTAATATGTTCTACAGTGTGG-3  size (162bp or longer) |
